# Myasthenia-Like Presentation of Imidacloprid Toxicity

**DOI:** 10.7759/cureus.35451

**Published:** 2023-02-25

**Authors:** Noel James, Tamilpavai Arulnambi, Parvathy K S, Nirumal Khumar, Lakshmi Narasimhan Ranganathan

**Affiliations:** 1 Neurology, Madras Medical College, Chennai, IND

**Keywords:** acetyl choline receptor, des-nitro imidacloprid, myasthenia, neonicotinoid, imidacloprid

## Abstract

Imidacloprid is a neonicotinoid insecticide highly specific to nicotinic acetylcholine receptors in insects and other invertebrates. Nicotinic receptors in mammalian species have a low affinity to neonicotinoids. However, cross-reactivity with mammalian nicotinic receptors is a major concern especially due to the propensity of this commonly used agent to persist in environmental water sources for an extended period of time. Here, we present a case report of a patient who presented to the emergency department with features suggestive of neuromuscular junction dysfunction, following exposure to imidacloprid.

## Introduction

Imidacloprid is a neurotoxic insecticide that belongs to a class of agents called neonicotinoids. It is commonly used to control insect pests in agriculture and nursery. It has structural and functional homology to nicotine [1] and it interferes with synaptic transmission between neurons and also at the neuromuscular junction. Neonicotinoids differ from nicotinoids in that there is selectivity towards insect nicotinic acetylcholine receptors compared to mammalian acetylcholine receptors [2]. It is one of the safest insecticidal agents in use because of its selective action on invertebrates and its effects on humans are limited to headaches, and rarely seizures [3]. Neuromuscular junction dysfunction in humans has not been reported as yet.

## Case presentation

**Figure 1:**
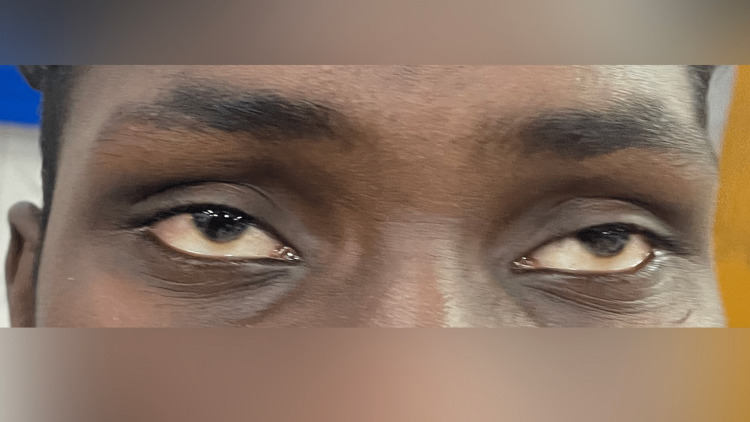
Asymmetric ptosis and worsening of ptosis on sustained upgaze

An 18-year-old boy, who works in his family's vegetable garden part-time, presented to the Emergency ward with a history of drowsiness and irritability since the past day. This was associated with double vision and drooping of both eyelids for the past 18 hours. On examination, extra-ocular movements were restricted in the form of bilateral abduction restriction, bilateral upgaze restriction, and worsening of ptosis on prolonged upgaze in less than a minute as depicted in Figure 1. There was no limb weakness, bulbar symptoms, respiratory insufficiency, or seizures. GCS was 15/15. He was found to have been involved in spraying Immidea (Imidacloprid) pesticide in his field two hours prior to developing symptoms. 3-Hz Repetitive nerve stimulation showed a decremental response of >10% in his right orbicularis oculi. The Serum AChR receptor antibody was negative. He was managed with benzodiazepines for his agitation and supportive care was given. He recovered over two days and was discharged.

## Discussion

Imidacloprid is an agonist at the nicotinic acetylcholine receptor at the neuronal and neuromuscular junctions in insects and vertebrates. Imidacloprid causes prolonged activation of the nicotinic acetylcholine receptor and leads to desensitization of the receptor. It is currently gaining widespread popularity due to its lethal effects on insects, with mammals relatively spared from its toxic effects [3]. Due to pest resistance, organophosphates and carbamates have higher mammalian toxicity and decreased effectiveness. The major degradation product of imidacloprid in the environment is desnitro-imidacloprid. This compound has poor penetration of the blood-brain barrier and only mild symptoms such as vomiting, diarrhea, headache, and abdominal pain are common in humans. Autonomic symptoms include hypertension and tachycardia, which require symptomatic management only in most cases. Rarely seizures, sedation, and respiratory arrest have been reported to be a cause of death [4]. Structural studies predict that the electron-deficient nitrogen atom of the imidazoline group of Imidacloprid may interact with the mammalian nicotinic AChR [5]. The metabolite des-nitro imidacloprid also has a nicotinic-type action (as opposed to neonicotinoid-type actions) and thus preferably affects mammalian nicotinic AChR when compared to insect nAChR [6]. Nicotinic acetylcholine receptor dysfunction due to either genetic or autoimmune pathology is associated with multiple pathogenic states including congenital myasthenia, myasthenia gravis, and frontal lobe dysfunction. Toxic insult to the nicotinic acetylcholine receptor due to the pertinent insecticide has also led to a clinical scenario characterized by fatigable weakness and ophthalmoplegia suggestive of neuromuscular junction dysfunction and irritability and agitation suggestive of cerebral dysfunction.

## Conclusions

This case as depicted above presented with features suggestive of neuromuscular junction dysfunction, that has not been reported in case reports previously. The rationale of neonicotinoid causing neuromuscular junction dysfunction and thereby respiratory insufficiency is appealing and given the emerging widespread popularity of neonicotinoids, further studies on the effects of these compounds on human neuromuscular junction need to be done. Emphasis on adequate use of protective gear has to be emphasized and as the compound is primarily a water contaminant, measures to avoid contamination of potable water sources have to be undertaken.
